# Transcriptomic analysis reveals the functions of H_2_S as a gasotransmitter independently of Cys in *Arabidopsis*

**DOI:** 10.3389/fpls.2023.1184991

**Published:** 2023-06-02

**Authors:** Huihui Fang, Zhenyuan Yu, Kehong Xing, Lingyi Zhou, Yuke Shao, Xiaofang Zhang, Yanxi Pei, Lu Zhang

**Affiliations:** ^1^ Key Laboratory of Quality and Safety Control for Subtropical Fruit and Vegetable, Ministry of Agriculture and Rural Affairs, Collaborative Innovation Center for Efficient and Green Production of Agriculture in Mountainous Areas of Zhejiang, College of Horticulture Science, Zhejiang Agriculture and Forestry University, Hangzhou, Zhejiang, China; ^2^ School of Life Science and Shanxi Key Laboratory for Research and Development of Regional Plants, Shanxi University, Taiyuan, Shanxi, China; ^3^ Zhejiang Provincial Key Laboratory of Bioremediation of Soil Contamination, College of Environment and Resources, College of Carbon Neutrality, Zhejiang Agriculture and Forestry University, Hangzhou, Zhejiang, China

**Keywords:** hydrogen sulfide, gasotransmitter, cysteine, transcriptome sequencing, *Arabidopsis*

## Abstract

Numerous studies have revealed the gasotransmitter functions of hydrogen sulfide (H_2_S) in various biological processes. However, the involvement of H_2_S in sulfur metabolism and/or Cys synthesis makes its role as a signaling molecule ambiguous. The generation of endogenous H_2_S in plants is closely related to the metabolism of Cys, which play roles in a variety of signaling pathway occurring in various cellular processes. Here, we found that exogenous H_2_S fumigation and Cys treatment modulated the production rate and content of endogenous H_2_S and Cys to various degrees. Furthermore, we provided comprehensive transcriptomic analysis to support the gasotransmitter role of H_2_S besides as a substrate for Cys synthesis. Comparison of the differentially expressed genes (DEGs) between H_2_S and Cys treated seedlings indicated that H_2_S fumigation and Cys treatment caused different influences on gene profiles during seedlings development. A total of 261 genes were identified to respond to H_2_S fumigation, among which 72 genes were co-regulated by Cys treatment. GO and KEGG enrichment analysis of the 189 genes, H_2_S but not Cys regulated DEGs, indicated that these genes mainly involved in plant hormone signal transduction, plant-pathogen interaction, phenylpropanoid biosynthesis, and MAPK signaling pathway. Most of these genes encoded proteins having DNA binding and transcription factor activities that play roles in a variety of plant developmental and environmental responses. Many stress-responsive genes and some Ca^2+^ signal associated genes were also included. Consequently, H_2_S regulated gene expression through its role as a gasotransmitter, rather than just as a substrate for Cys biogenesis, and these 189 genes were far more likely to function in H_2_S signal transduction independently of Cys. Our data will provide insights for revealing and enriching H_2_S signaling networks.

## Graphical Abstract

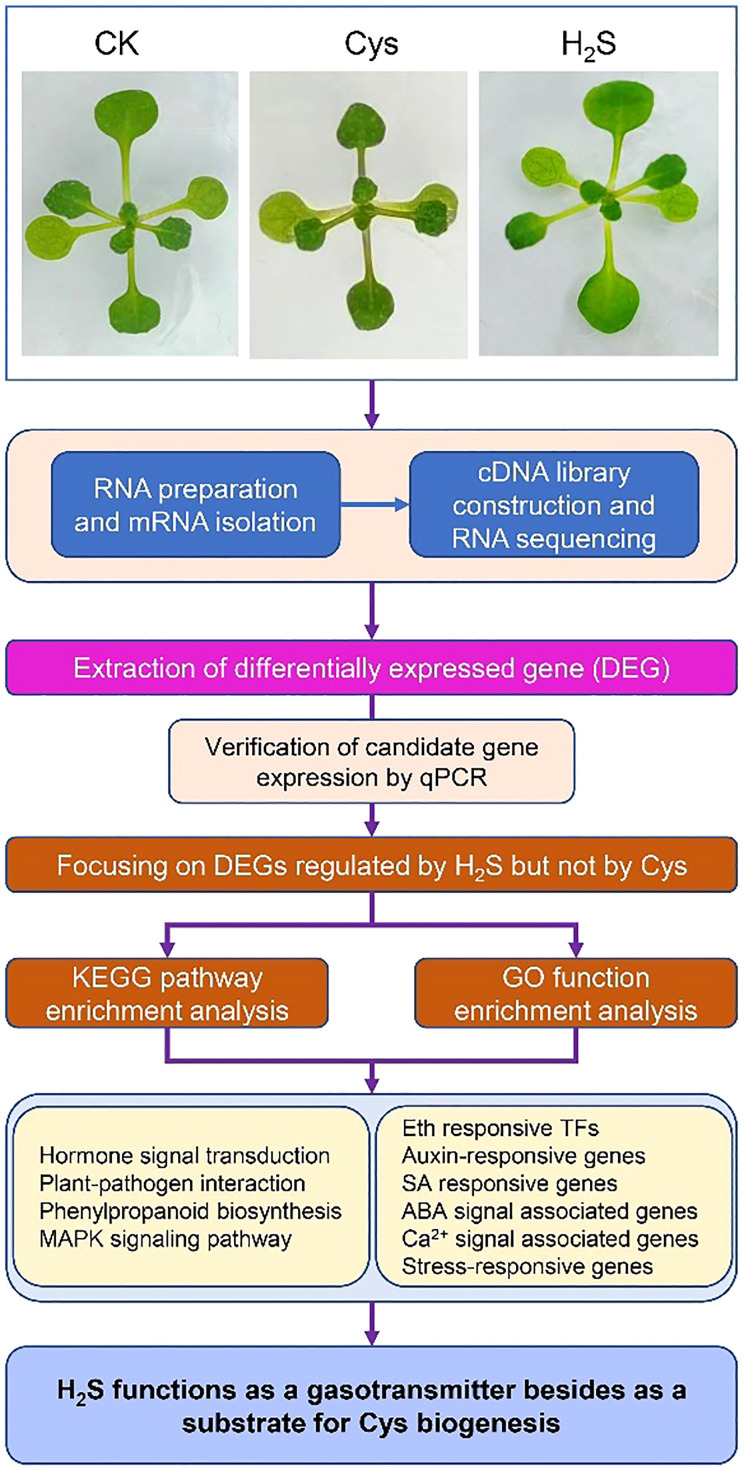

## 1 Introduction

Following the original paper postulating the novel concept of “gasotransmitter” nearly 20 years ago, the importance of these gaseous signaling molecules in biological pathways has been widely reported (Wang, 2002; Wang, 2014; Feng et al., 2019; Corpas and Palma, 2020; Aroca et al., 2021; Liu et al., 2021; Yang et al., 2022). Hydrogen sulfide (H_2_S) is qualified as the third member of gasotransmitter family following the identification of nitric oxide and carbon monoxide (Wang, 2002; Yang et al., 2008). For hundreds of years since its discovery, H_2_S has been well-known as a gas with an unpleasant odor and high toxicity (Fu et al., 2018; Lefer, 2019). Even the occasional discovery that H_2_S can increase plant resistance to pests and pathogens has been attributed to the cytotoxic properties of H_2_S (Wang, 2012). Until recent decades, the endogenously generation of H_2_S in animals and plants reignited scientists’ thinking on the significance of endogenous H_2_S in organism.

The generation of endogenous H_2_S in plants is closely related to Cys metabolism, and it can be roughly summarized into two pathways, one is that Cys desulfhydrases (CDes) catalyze the degradation of Cys to produce H_2_S, and the other is that H_2_S is generated as a side reaction product during *O*-acetylserine(thiol)lyase (OAS-TL) mediated Cys biosynthesis (Papenbrock et al., 2007; Heeg et al., 2008; Alvarez et al., 2010). For the former pathway, L/D-CDes (L-CDes; EC 4.4.1.28 and D-CDes; EC 4.4.1.15), requiring pyridoxal 5’-phosphate (PLP) as a cofactor, primarily responsible for the generation of endogenous H_2_S in plant, catalyze the desulfuration of Cys to H_2_S plus ammonia and pyruvate in a stoichiometric ratio of 1:1:1 (Jin and Pei, 2015; Liu et al., 2021). There were some CDes genes have been reported in *Arabidopsis*, including L-Cys desulfhydrase (LCD, AT3G62130), D-Cys desulfhydrase 1 (DCD1, AT1G48420) and D-Cys desulfhydrase 2 (DCD2, AT3G26115) (Papenbrock et al., 2007). Specially, the L-Cys desulfhydrase 1 (DES1, AT5G28030), identified from a previously reported minor cytosolic OAS-TL protein CS-LIKE, is demonstrated as a novel L-Cys desulfhydrase (Alvarez et al., 2010). DES1 and LCD using L-Cys as the substrate are the most widespread in plants. The substrate of DCD1 is D-Cys, while DCD2 can degrade the two isomers of Cys. In addition, another Cys desulfuration reaction occurs in Fe-S cluster biosynthesis and involves the formation of L-Ala and elemental sulfur or H_2_S from Cys through an enzyme-bound persulfide (-SSH) intermediate, which now has been known catalyzed by NifS-like proteins (Zheng and Dean, 1994; Rydz et al., 2021). In Arabidopsis, *AtNFS1* (*AT5G65720*) encodes a cysteine desulfurase and AtNFS2 (AT1G08490) is a chloroplastic NifS-like protein, and both AtNFS1 and AtNFS2 require PLP as cofactor for proper folding. H_2_S can be produced with the availability of an appropriate amount of reducing agent to provide electrons during AtNFS1 and AtNFS2 mediating the Fe-S clusters formation (Leon et al., 2002; Pilon-Smits et al., 2002). For the latter pathway, H_2_S can be generated as a side product of Cys biosynthesis, accomplished by two sequential reactions catalyzed by Ser acetyltransferase (SAT; EC 2.3.1.30) and OAS-TL (EC 2.5.1.47). OAS-TL catalyzes the incorporation of sulfide (i.e., H_2_S) to *O*-acetyl-serine (OAS) to induce the last step of Cys synthesis (Romero et al., 2014), which is a reversible reaction where Cys could be decomposed to H_2_S and OAS (Gotor et al., 2019; Sehar et al., 2022). In Arabidopsis, the *OAS-A1* (*AT4G14880*), *OAS-B* (*AT2G43750*), and *OAS-C* (*AT3G59760*) encode authentic OAS-TL proteins located in the cytosol, plastids, and mitochondria of Arabidopsis cells, respectively (Wirtz et al., 2004; Wirtz and Hell, 2006). The *CYS-C1* (*AT3G61440*) encodes another OAS-TL isoform, which actually functions as the *β*-cyanoalanine synthase (β-CAS; EC 4.4.1.9) and catalyzes the reaction between L-Cys and HCN to synthesize β-cyanoalanine and H_2_S, a process that linking cyanide detoxification and H_2_S generation (Alvarez et al., 2012; Birke et al., 2012; Fang et al., 2022). The remaining OAS-TL-like proteins encoding genes include *CYS-C1* (*AT3G61440*), *CYS-D1* (*AT3G04940*), *CYS-D2* (*AT5G28020*), *DES1* (*AT5G28030*), and *CS26* (*AT3G03630*) (Yamaguchi et al., 2000). It’s worth noting that DES1, mentioned above, is an OAS-TL homology but has nonconservative amino acid changes in its β8A-β9A loop, an important structure for interaction with SAT, so DES1 can not interact with SAT to mediate Cys biosynthesis and has more than 10 times lower affinity for OAS as a substrate than that for L-Cys, therefore, DES1 has been confirmed as a L-Cys desulfhydrase to produce endogenous H_2_S in plants (Alvarez et al., 2010). Coherently, H_2_S generation occurs during the regulation of Cys homeostasis, a process that Cys synthesis and degradation in the cell are coordinated mainly through the activities of OAS-TL and CDes.

H_2_S fulfills all criteria for being a gasotransmitter, such as being a gaseous molecule, generated endogenously, small and generally reactive, exerting important signaling roles. Evidence has been accumulating to elucidate the physiological functions of H_2_S in numerous physiology processes from the perspective of gaseous signaling molecule (Lu et al., 2013; Wang, 2014; Aroca et al., 2021; Li et al., 2022; Yang et al., 2022), including crosstalk with phytohormone signals (Jia et al., 2018; Shen et al., 2020; Zhou et al., 2021), interaction with Ca^2+^ signaling (Fang et al., 2017) and NO signals (Lu et al., 2013; Liu et al., 2021), regulation of gene transcription, and mediation of protein modification (Paul and Snyder, 2015; Aroca et al., 2018; Ma et al., 2021), etc. Mediating protein S-persulfidation, converting the mercapto groups (-Cys-SH) into hydropersulfide groups (-Cys-SSH), has been confirmed to be a key route for H_2_S bioactivity and signaling transduction, and involves in a myriad of cellular processes in plants linked to growth, development, stress responses, and phytohormone signals (Aroca et al., 2018; Shen et al., 2020; Ma et al., 2021; Sun et al., 2021). Nonetheless, there are still a few objections that the positive functions of H_2_S just due to its role as a substrate for S metabolism, especially for the synthesis of Cys, which is the first organic compound containing reduced S synthesized by the plants (Takahashi et al., 2011; Romero et al., 2014). Cys occupies a central position in plant metabolism because it per se and its derivative molecules play roles in a variety of signaling pathway occurring in various cellular processes (Paulsen and Carroll, 2013; Romero et al., 2014). Therefore, the controversy centers on the possibility that H_2_S acts only as a substrate to promote the synthesis of Cys or endogenous H_2_S production disturbs the Cys homeostasis, and the H_2_S-induced Cys, not H_2_S itself, is the core molecule that plays the role of signal transduction.

In this study, we provide some evidence through comprehensive transcriptome analysis to support the conclusion that H_2_S functions as a gasotransmitter, besides as the substrate for Cys biogenesis, providing insights for revealing and enriching H_2_S signal transduction networks.

## 2 Materials and methods

### 2.1 Plants and treatments

The Columbia wild type *Arabidopsis thaliana* (Col-0) was used in this study, and the seeds were surface sterilized by soaking in 70% ethanol for 5 min and rinsed well with sterile distilled water (3 times). These surface sterilized seeds were planted on 1/2 Murashige and Skoog (½MS) solid medium with 0.7% (w/v) agar and 1.5% sucrose. After 2 days stratification in darkness at 4°C, seeds were transferred to plant incubators with a 10 h-light (130 μmol m^-2^ s^-1^)/14 h-dark photoperiod at 22°C.

For H_2_S fumigation treatment, the 7-day-old seedlings were transferred aseptically to ½MS medium and successively fumigated with H_2_S released by NaHS. The NaHS solution-containing tube was placed in the Petri dish and the H_2_S fumigation concentration was 50 μmol L^-1^ (the volume was calculated by subtracting the volume of culture medium from the total volume of Petri dish). For Cys treatment, the 7-day-old seedlings were transferred aseptically to ½MS containing 1 mmol L^-1^ Cys, and the Cys containing ½MS was prepared by directly adding corresponding volume of Cys mother liquor, sterilized by filtration, to the ½MS medium. In order to enhance the scientific credibility of the one single variable principle, the 7-day-old seedlings were also transferred aseptically to ½MS medium for the control.

After 10 days of treatment, the seedling phenotypes were observed, and the whole plants were taken for subsequent anthocyanin level analysis, the determination of endogenous H_2_S and Cys contents as well as the enzymatic activities. The aerial tissue of Arabidopsis seedlings was obtained for RNA preparation and transcriptome sequencing.

### 2.2 Determination of the enzymatic activities of CDes and OAS-TL

Aliquots of 500 mg of seedlings were powdered in liquid nitrogen for small-scale extraction of proteins, and the power was dissolved in 0.5 mL of extraction buffer (50 mmol L^-1^ HEPES-KOH, pH 7.4, 10 mmol L^-1^ KCl, 1 mmol L^-1^ EDTA, 1 mmol L^-1^ EGTA, 10% [v/v] glycerin, 10 mmol L^-1^ DTT, and 0.5 mmol L^-1^ phenylmethylsulfonyl fluoride [PMSF]). The proteins were extracted at 4°C for 1 hour with frequent shaking, and the centrifugation at 4°C for 10 min and 12,000 g was performed to get the supernatant containing total proteins, which were then used to detect the enzymatic activity (Heeg et al., 2008).

The OAS-TL proteins catalyze the last step of Cys biosynthesis, so the enzymatic activity of OAS-TL was detected to represent the intensity of Cys production in plants. The enzymatic activity of OAS-TL was determined according to method reported previously (Liu et al., 2019). Briefly, the protein extraction was added to a reaction mixture of 50 mmol L^-1^ Tris-HCl (pH 7.5), 5 mmol L^-1^ DTT, 5 mmol L^-1^ OAS and 5 µmol L^-1^ PLP. The reaction was initiated by adding 10 mmol L^-1^ Na_2_S. After incubating for 10 min at 25°C, the reaction was terminated by adding 100 µL absolute acetic acid. Then, 200 µL ninhydrin reagent (25 mg/ml in acetic acid:HCl, 60:40, v/v) was added to detect the generated Cys. The mixture was boiled for 10 min before being cooled rapidly and 200 µL absolute ethanol was added to stop the reaction. Finally, the absorbance of the reaction was measured at 560 nm to determine the cysteine concentration.

The CDes are the most important enzyme that catalyzes the decomposition of Cys to pyruvate, ammonia, and H_2_S, so the activity of CDes was ascertained by measuring the production rate of H_2_S from Cys (Fang et al., 2017). The enzyme activity was determined in a reaction mixture (1 mL) containing 100 mmol L^-1^ Tris-HCl (pH 9.0), 0.8 mmol L^-1^ L-Cys or D-cysteine, 2.5 mmol L^-1^ DTT and 100 µL protein extraction. The reaction was incubated at 37°C for 15 min, and the generated H_2_S was absorbed by 0.5 mL Zn(AC)_2_ when placed together in a closed vial with the aforementioned reaction mixture. Then, 100 µL of 20 mmol L^-1^ N, N-dimethyl-p-phenylenediamine and 100 µL of 30 mmol L^-1^ FeCl_3_ were added to the Zn(AC)_2_ solution. After incubation in dark for 15 min, the absorbance was measured at 670 nm to determine the level of produced H_2_S.

### 2.3 Measurement of the contents of endogenous Cys and H_2_S

The endogenous H_2_S content was measured according to previously described method based on the principle that H_2_S reacts with N, N-dimethyl-p-phenylenediamine in the presence of the FeCl_3_ to produce blue methylene blue, which has the maximum absorption at 670 nm (Qiao et al., 2015; Fang et al., 2016). Cys can react specifically with acid ninhydrin to form a pink product, which has a maximum absorbance at 560 nm. The reaction is highly sensitive for Cys determination, so the Cys content was determined according to the method based on this reaction (Gaitonde, 1967; Fang et al., 2016).

### 2.4 Analysis of anthocyanin level

Anthocyanin content was measured as described previously (Rabino and Mancinelli, 1986). Briefly, 100 mg Arabidopsis seedlings were incubated in 1 mL extraction buffer (methanol containing 1% HCl, v/v) for 24 h in darkness at 4°C with occasional shaking. After extraction, the mixture was then centrifuged for 15 min at 5000 rpm, and then the supernatants were collected to detect the absorbance at 530 and 657 nm. The concentration of the anthocyanin was presented as mg g^-1^ dry weight of the differently treated plants using the following equation: [OD_530_-0.25*OD_657_] * volume of the extract (mL)/fresh weight (g).

### 2.5 RNA extraction and library preparation for transcriptome sequencing

The RNA extraction, library preparation and transcriptome sequencing were performed by Beijing Biomarker Biotechnology Co., Ltd., Beijing, China. Briefly, total RNA was isolated from seedlings using RNAiso-plus (TaKaRa), and the total RNA was analyzed by NanoDrop 2000 (Thermo Fisher Scientific, Wilmington, DE) for concentration and purity, and by the RNA Nano 6000 Assay Kit of the Agilent Bioanalyzer 2100 (Agilent Technologies, CA, USA) for RNA integrity. Then 1 μg total RNA was used as input to prepare the non-strand-specific RNA-seq library by using NEBNext UltraTM RNA Library Prep Kit for Illumina (NEB, USA) and index codes were added to attribute sequences to each sample following manufacturer’s recommendations. Briefly, mRNA was purified from total RNA by using poly-T oligo-attached magnetic beads, and then fragmented using divalent cations under elevated temperature in NEBNext First Strand Synthesis Reaction Buffer (5X). Subsequently, first strand cDNA was synthesized using random hexamer primer and M-MuLV Reverse Transcriptase, and second strand cDNA synthesis was then performed using DNA Polymerase I and RNase H. Remaining overhangs were converted into blunt ends *via* exonuclease/polymerase activities. After adenylation of 3’ends of DNA fragments, NEBNext adaptor with hairpin loop structure were ligated to prepare for hybridization. The library fragments were purified with AMPure XP system (Beckman Coulter, Beverly, USA) to select cDNA fragments of preferentially 240 bp in length. With these operations, we generated the size-selected and adaptor-ligated cDNA, which was then incubated with 3 μL of USER Enzyme (NEB, USA) at 37°C for 15 min followed by 5 min at 95°C before PCR. Then, library amplification was performed with PCR using Phusion High-Fidelity DNA polymerase, universal PCR primers and index (X) primer. Finally, PCR products were purified (AMPure XP system) and library quality was assessed on the Agilent Bioanalyzer 2100 system.

After cluster generation of the index-coded samples, which was performed on a cBot Cluster Generation System using TruSeq PE Cluster Kit v4-cBot-HS (Illumia), the library sequencing was carried out on an Illumina platform.

### 2.6 Data processing and analysis

After sequencing, the paired-end raw reads were generated, and the raw reads were firstly processed through in-house perl scripts to remove low quality reads, adapter containing reads, ploy-N containing reads. Then, Q20, Q30, GC-content and sequence duplication level of the clean data were calculated. All the downstream analyses were based on clean data with high quality. Only clean reads with a perfect match or one mismatch were further analyzed and annotated based on the reference genome. Hisat2 tools soft were used to map with reference genome. Gene function was annotated based on the following databases: Nr (NCBI non-redundant protein sequences); Nt (NCBI non-redundant nucleotide sequences); Pfam (Protein family); KOG/COG (Clusters of Orthologous Groups of proteins); Swiss-Prot (A manually annotated and reviewed protein sequence database); KO (KEGG Ortholog database); GO (Gene Ontology).

### 2.7 Quantification of gene expression levels and differential expression analysis

Gene expression levels were estimated by FPKM. Differential expression analysis of two conditions/groups was performed using the edgeR. The resulting *P* values were adjusted using the Benjamini and Hochberg’s approach for controlling the false discovery rate. Genes with an adjusted *P*-value < 0.05 found by edgeR and Fold Change >1.5 were assigned as differentially expressed.

### 2.8 GO enrichment analysis

GO enrichment analysis of the DEGs was implemented using the GOseq R packages based Wallenius non-central hyper-geometric distribution, which can adjust for gene length bias in DEGs (Young et al., 2010).

### 2.9 KEGG enrichment analysis

KEGG (Kanehisa et al., 2008) is a database resource for understanding high-level functions and utilities of the biological system, such as the cell, the organism and the ecosystem, from molecular-level information, especially large-scale molecular datasets generated by genome sequencing and other high-throughput experimental technologies (http://www.genome.jp/kegg/). The statistical enrichment of differential expression genes in KEGG pathways was analyzed by using KOBAS software (Mao et al., 2005).

### 2.10 Protein-protein interaction analysis

PPI is the basis on which many biological pathways are built, so PPI prediction is essential for understanding the function mechanism of proteins. DEGs were blast (blastx) to the genome of a related species (the protein-protein interaction of which exists in the STRING database: http://string-db.org/) to get the predicted PPI of these DEGs. Then the PPI networks of these DEGs were visualized in Cytoscape (Shannon et al., 2003).

### 2.11 Validation of transcriptomic data by qRT-PCR

Total RNA was isolated from seedlings by using RNAiso-plus (TaKaRa, Shiga, Japan, Cat9109) according to the manufacturer’s instructions. The cDNA was synthesized using a reverse transcription system kit (PrimeScript RT Reagent Kit, TaKaRa, RR037B) and oligo (dT) primers, and then the qRT-PCR was performed to validate the transcriptomic data of target genes according to the instructions of the Bio-Rad Real-Time System (CFX96TM C1000 Thermal Cycler). In our study, the *UBQ4* (*AT5G20620*), a housekeeping reference gene, was used as the normalizer. All of the primer pairs used for qRT-PCR were checked for amplification specificity and were listed in **Table 1**.

**Table 1:** List of all primers for qRT-PCR used in this study.

All of the primer pairs used for qRT-PCR in this study		
Genes	Accession Number	Primer pairs (5’-3’)
*UBQ4*	*AT5G20620*	F, GGGCACTCAAGTATCTTGTTAGC
		R, TGCTGCCCAACATCAGGTT
*BON1*	*AT5G61900*	F, ACATTGGTGTTTCGTGTGTATG
		R, GAGTTCTAATGTGCTCGTCCTA
*BAP1*	*AT3G61190*	F, ATCAAGAAAAAGACTTTCGCCG
		R, GATTGCTTCTTCTCGATTCGTC
*ZAT10*	*AT1G27730*	F, TTCTTCAGTCTTCCATGGAGTC
		R, CGAGAGCTTGGTAAGAAGAGAA
*ZAT12*	*AT5G59820*	F, TGTCGTCTGGATTGATGAAGAA
		R, GATTTCTTCAACGTAGTCACCG
*CAF1-9*	*AT3G44260*	F, CTCAATGGACACAGAATTTCCC
		R, CGTCGACGTTAGCTTTAAGAAG
*GSTF2*	*AT4G02520*	F, GTATCAAAGTTTTCGGACACCC
		R, TCCTTGGTTTTCATATCGGTGA
*GSTF7*	*AT1G02920*	F, TGAAGATGGAGACTTCAAGCTT
		R, CATGCGATTCAATTTCAATGCC
*GSTF6*	*AT1G02930*	F, TAGCCAAAGTCCTCGATGTTTA
		R, GAAGATCGACCAAAGTGAAGTG
*LSU3*	*AT3G49570*	F, GAGGTCGAGTCTTTAGATCAGG
		R, TCGTAACAACGACTTCAAGAGA
*SDI1*	*AT5G48850*	F, GGAAGATTGGTTCTTGACGATG
		R, CTCTTCAAGCCCAAGAACAAAG
*LSU2*	*AT5G24660*	F, GGGAAAGGAGGAAACTATGTGA
		R, AGCTCGTTCATGAGAAAGATGA
*GGCT2;1*	*AT5G26220*	F, GTACTCCTGAACATCCTGCTAG
		R, CACTCTCTTCGTTCCAAGTACT
*CRRSP38*	*AT3G22060*	F, GCGACAGGAGAGAAAAATATCG
		R, CAGTTAGGAAGCTCCCCAATAA
*CRRSP50*	*AT5G48540*	F, CACGAGAGACTTAAGCGAATTG
		R, AATTCTACCAACACTAGGACCG
*PRX70*	*AT5G64110*	F, AGGGACAGATTCTTCAACTACG
		R, AGGTACGACGTATCAAATTGGT
*PRX71*	*AT5G64120*	F, CTAGAGCTGAGACTATTGTCCG
		R, GTTTTGGCGTTGTCTATGACTT
*CHS*	*AT5G13930*	F, TCTTTGGATGAGATCAGACAGG
		R, GCGGAAGTAGTAGTCAGGATAC
*CHI*	*AT3G55120*	F, GGTAAATTCGTGATCTTCACCG
		R, TGTTAGCTCCTCCGTAGTTTTT
*F3H*	*AT3G51240*	F, ACTCGAGCAGATTATCCATAGC
		R, CTTATACATCTCGGCAAACGTG
*DFR*	*AT5G42800*	F, CTTCTTATACGAACAAGCAGCC
		R, TGAAGGTACGTTATATTCGGGG
*LDOX*	*AT4G22880*	F, CTGATTCGATTGTGATGCACAT
		R, ACAATCTTATCCTTTGGGGGTT
*UF3GT*	*AT5G54060*	F, CGAGACCATTTTCCGTACAATC
		R, CTAGAGGCGTCTTAGCTAACTC

## 3 Results

### 3.1 Effects of H_2_S and Cys treatment on seedling development in *Arabidopsis*

**Figure 1:**
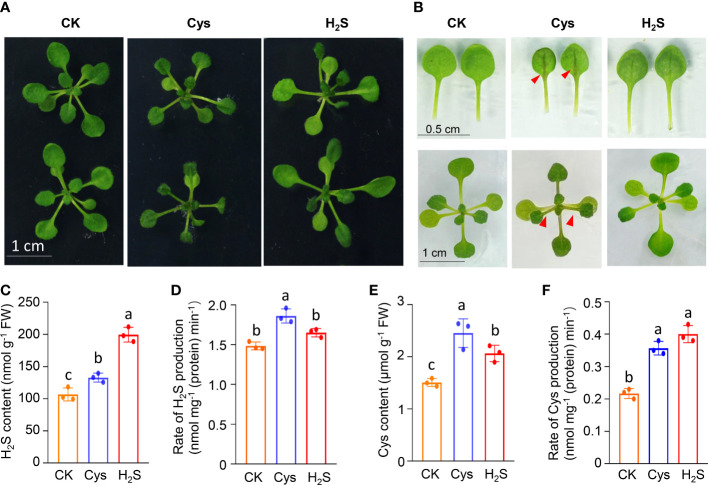
Responses of seedling development as well as endogenous H_2_S and Cys generation to exogenous H_2_S and Cys treatment in Arabidopsis. **(A)** Phenotypes of Arabidopsis seedlings after H_2_S and Cys treatment; **(B)** Leaf size and anthocyanidin color in leaves of H_2_S fumigated and Cys treated seedlings. **(C, E)** Endogenous H_2_S and Cys content in H_2_S fumigated and Cys treated seedlings. **(D, F)** Production rate of endogenous H_2_S and Cys in H_2_S fumigated and Cys treated seedlings. Red triangle indicates the anthocyanin accumulation in the center vein of leaves in Cys treated seedlings. Different letters represent statistically significant differences (*p* < 0.05).

**Figure 2:**
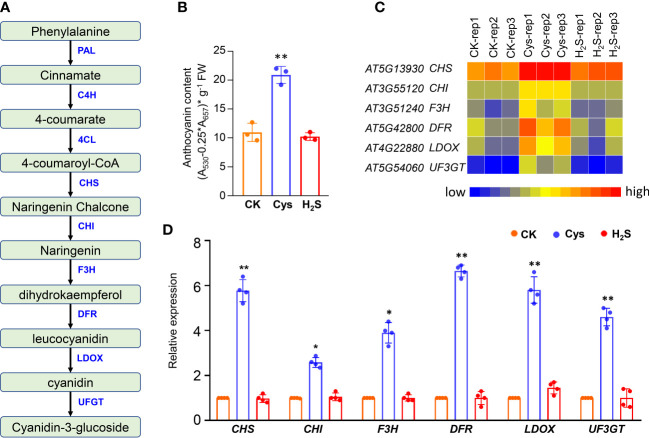
Analysis of anthocyanin level and anthocyanin biosynthesis genes in H_2_S and Cys treated seedlings. **(A)** Schematic representation of anthocyanin biosynthesis in plants. **(B)** Anthocyanin content of H_2_S and Cys treated seedlings. **(C, D)** Heatmap representation **(C)** and qRT-PCR analysis **(D)** of the anthocyanin biosynthesis genes (*CHS*, *CHI*, *F3H*, *DFR*, *LDOX*, *UF3GT*) in H_2_S and Cys treated seedlings. The symbol * indicates significant difference at the 0.05 level (*p* < 0.05) and ** indicates significant difference at the 0.01 level (*p* < 0.01).

To analyze the correlation and distinction of exogenous H_2_S and Cys treatment on seedling development, we observed and compared the Arabidopsis seedlings phenotypes after 10 days of 50 μmol L^-1^ of H_2_S fumigation and 1 mmol L^-1^ of Cys treatment. It was found that no obvious stress phenotypes were produced in H_2_S and Cys treated seedlings (**Figure 1A**). Exogenous H_2_S fumigation had little effect on the relative speed of plant growth, while Cys treatment seemed to cause a slower leaf growth rate of seedlings with smaller leaves at the same days after planting (**Figure 1A**). Anthocyanins are natural water-soluble pigments extensively exist in plants, and the anthocyanin level in leaves provides valuable information about the physiological status of plants. In our study, we found that Cys treatment, but not H_2_S fumigation, could lead to darker red and purple colors in leaves, especially in the petioles and center veins of leaves (**Figure 1B**). Moreover, analysis of anthocyanin content indicated that the anthocyanin level of the leaves was increased by Cys treatment but not affected by H_2_S fumigation (**Figure 2B**), which was consistent with the phenotype that darker anthocyanin color in leaves of Cys-treated seedlings. All these results mentioned above indicated the specificity of H_2_S and Cys in regulating seedling development.

### 3.2 Responses of endogenous H_2_S and Cys generation to exogenous H_2_S and Cys treatment

Exogenous application of signaling molecule could induce endogenous signal, so we detected the content and production rate of endogenous H_2_S and Cys in exogenous H_2_S and Cys treated seedlings. The data showed that both H_2_S fumigation and Cys treatment could simultaneously increase the content of endogenous H_2_S and Cys to a certain degree (**Figures 1C, E**), suggesting that the two kinds of exogenous treatments in our study activated both the endogenous H_2_S and Cys signals to various degrees. Our data also demonstrated that exogenous H_2_S and Cys treatment regulated the endogenous Cys-H_2_S cycle by modulating the production intensity of endogenous H_2_S and Cys. Both the production rate of H_2_S (**Figure 1D**), mediated by Cys degradation, and the production rate of Cys (**Figure 1F**) could be enhanced by Cys treatment, indicating that Cys treatment promoted the cyclic metabolism of Cys-H_2_S. However, H_2_S fumigation didn’t affect the production rate of H_2_S (**Figure 1D**), suggesting that the CDes activities and endogenous H_2_S generation were not activated in H_2_S fumigated seedlings. Because of the property of small molecular gas, H_2_S can be directly uptaken by leaves, so our data suggested that the increase of endogenous H_2_S content in seedlings fumigated by H_2_S probably due to the direct uptake of H_2_S by the leaves, rather than stimulating CDes mediated endogenous H_2_S production. The increased production rate of Cys in H_2_S fumigated seedlings (**Figure 1F**) might because of the higher level of endogenous H_2_S content.

### 3.3 Overview of the transcriptome profiles in Cys and H_2_S treated seedlings

**Figure 3:**
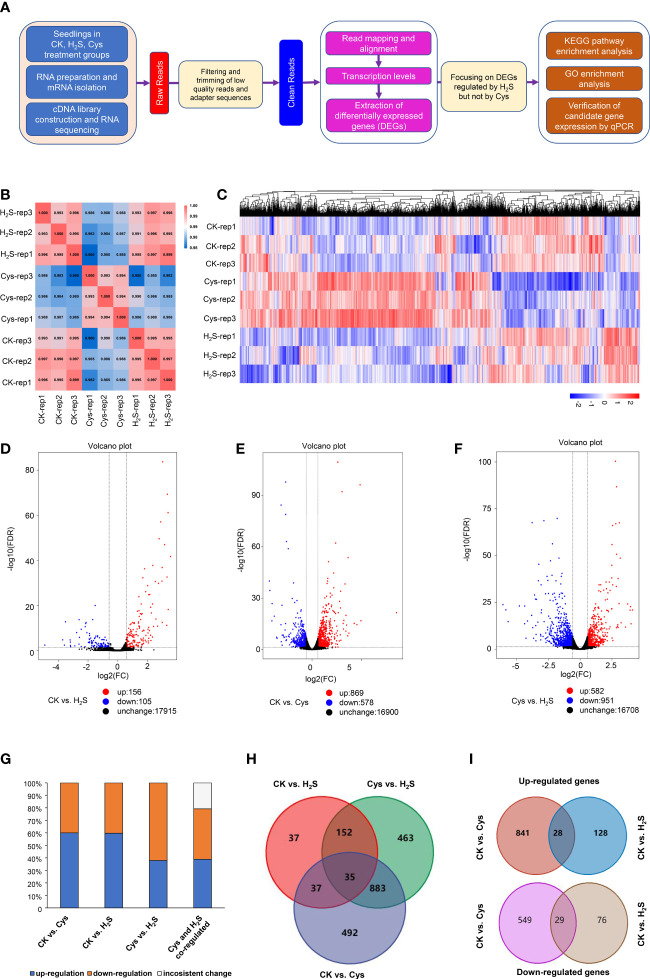
Characterization of transcriptome profiles in seedlings responding to H_2_S and Cys treatment. **(A)** Flow chart of analyzing transcriptome sequencing data. **(B)** Heat diagram of correlation coefficient between the 9 mRNA samples. **(C)** Heatmap representation of a one-dimensional hierarchical clustering of DEGs as determined by mRNA sequencing. **(D–F)** Volcano Plot showing the number of differentially expressed mRNAs (FDR < 0.05 and Fold Change >1.5) in different compared groups, **(D)** CK vs. H_2_S, **(E)** CK vs. Cys, **(F)** Cys vs. H_2_S. **(G)** Percentage of up-regulated and down-regulated genes in Cys-regulated, H_2_S-regulated, as well as H_2_S and Cys co-regulated DEGs. **(H)** Venn diagram showing the overlapping of DEGs in different compared groups, CK vs. Cys, CK vs. H_2_S, and Cys vs. H_2_S. **(I)** Venn diagram showing the overlapping of up-regulated DEGs and down-regulated DEGs in the comparison groups of CK vs. Cys and CK vs. H_2_S.

To reveal the correlation and distinction between H_2_S as a gasotransmitter and as a substrate for Cys synthesis, we performed comprehensive transcriptomic analysis based on RNA sequencing data of 9 libraries generated from RNA samples of Control (CK), H_2_S fumigated (H_2_S), and Cys treated (Cys) Arabidopsis seedlings with three replicates. The library construction and RNA sequencing data analysis flow chart was briefly summarized in the **Figure 3A**. We obtained a total of 57.76 Gb clean data, and the clean data of each library reached at least 5.81 Gb with average base quality (Q30) more than 92.36% (**Table S1**) and sample correlation coefficients more than 98% (**Figure 3B**), which indicated that the quality and accuracy of sequencing data were sufficient and reliable for subsequent analysis although the sampling method probably caused slight variation in replications of the same treatment. The reads of each sample were mapped to the designated reference genome, and the alignment efficiency ranged from 96.18% to 97.61% (**Table S1**), so the selected reference genome can meet our needs for information analysis.

### 3.4 Analysis of differentially expressed genes between H_2_S and Cys treated seedlings

Gene expression profiles calculated based on the fragments per kilobase of transcript per million fragments mapped (FPKM) value showed that there was no significant difference in the overall FPKM expression levels of transcripts among CK, H_2_S, Cys treated seedlings (**Figures S1A, B**). The principal component analysis (PCA) plot was used to provide a systematic view of the consistency and variance of our RNA sequencing dataset, and the result showed that the CK, Cys and H_2_S groups could be distinguished (**Figure S1C**). Furthermore, we identified the DEGs according to the gene expression profiles in H_2_S fumigated and Cys treated seedlings. EdgeR was used to normalize the data and extract DEGs with FDR < 0.05 and Fold Change >1.5. The Heatmap represented the one-dimensional hierarchical clustering of differential gene expression among these three groups (**Figure 3C**). The volcanic maps indicated that a total of 261 DEGs were identified in H_2_S fumigated seedlings, of which 156 genes (59.77%) were upregulated and 105 genes (40.23%) were downregulated (**Figures 3D, G**). Moreover, among these H_2_S-regulated genes, 72 genes were co-regulated by Cys treatment (**Figure 3H**), hinting that H_2_S might regulate the transcription of the other 189 genes through its role of gasotransmitter rather than as the substrate for Cys synthesis. There were 1447 DEGs caused by Cys treatment, including 869 (60.06%) upregulated genes and 578 (39.94%) downregulated genes (**Figures 3E, G**). Among these Cys-regulated genes, only 4.9% (72) of these DEGs could also respond to H_2_S fumigation and up to 95.1% (1375) of the genes could be regulated only by Cys treatment (**Figure 3H**), suggesting that Cys might play its role in regulating the expression of these genes through itself or its derivative molecules, bypass degrading to generate endogenous H_2_S. In the Cys and H_2_S co-regulated genes, 28 genes were co-up-regulated and 29 genes were co-down-regulated in Cys-treated and H_2_S-fumigated seedlings (**Table 2**, Sheet 2 and 3 of **Supp Excel S5**, and **Figure 3I**), however, 9 genes up-regulated by Cys treatment were down-regulated by H_2_S-fumigation, and 6 genes down-regulated by Cys treatment were up-regulated by H_2_S-fumigation (Sheet 4 of Supp Excel S5 and **Figure 3G**). We also identified 1533 DEGs between H_2_S-fumigated and Cys-treated seedlings, including 582 (37.96%) up-regulated genes and 951 (62.04%) down-regulated genes (**Figures 3F, G**). Consequently, H_2_S and Cys could induce different DEGs in Arabidopsis seedlings, indicating that H_2_S performed its functions not only by serving as a substrate of Cys but also by participating in other physiological pathways, possibly involving its gasotransmitter functions.

**Table 2:** List of genes co-up-regulated and co-down-regulated by H_2_S fumigation and Cys treatment.

List of 28 genes co-up-regulated by H_2_S fumigation and Cys treatment	List of 29 genes co-down-regulated by H_2_S fumigation and Cys treatment
*AT1G09070*, *AT1G19180*, *AT1G22190*, *AT1G25400*, *AT1G25560*, *AT1G32920*, *AT1G66180*, *AT1G72140*, *AT1G73500*, *AT1G76650*, *AT2G27080*, *AT2G27830*, *AT3G20370*, *AT3G49570*, *AT3G50800*, *AT4G23870*, *AT4G32480*, *AT4G37260*, *AT5G07580*, *AT5G14730*, *AT5G24660*, *AT5G26220*, *AT5G26260*, *AT5G37770*, *AT5G48850*, *AT5G61590*, *AT5G66650*, *AT5G67300*,	*AT1G51790*, *AT1G51800*, *AT1G51850*, *AT2G19190*, *AT2G25470*, *AT2G39200*, *AT2G39210*, *AT2G39518*, *AT2G43620*, *AT2G44370*, *AT3G22060*, *AT3G26230*, *AT3G46280*, *AT3G51440*, *AT4G11890*, *AT4G12470*, *AT4G12490*, *AT4G12500*, *AT4G14400*, *AT4G20000*, *AT5G01550*, *AT5G44575*, *AT5G44700*, *AT5G45570*, *AT5G48540*, *AT5G51480*, *AT5G64110*, *AT5G64120*, *AT5G67450*

### 3.5 Function enrichment analysis of H_2_S but not Cys regulated DEGs

**Figure 4:**
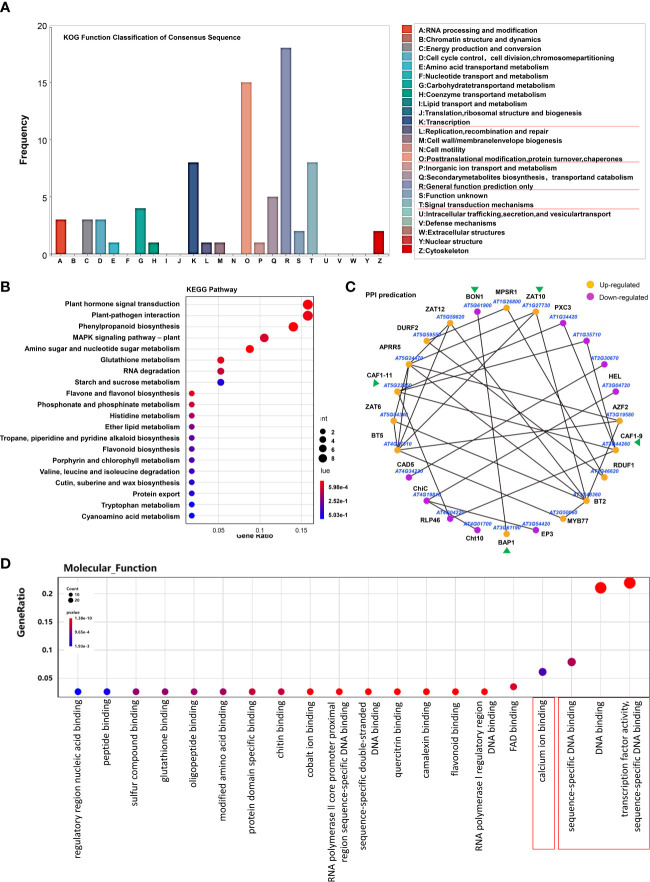
Overall analysis of the DEGs regulated by H_2_S fumigation but not by Cys treatment. **(A, B)** KOG function classification **(A)** and KEGG pathway **(B)** of the 189 DEGs that could be regulated by H_2_S fumigation but not by Cys treatment. **(C)** PPI network of proteins encoded by these DEGs. **(D)** GO enrichment analysis of these 189 DEGs.

In order to further explore the functions of H_2_S as a gasotransmitter, we then focused on these 189 genes regulated only by H_2_S fumigation but not by Cys treatment, including 122 up-regulated genes and 67 down-regulated genes. KOG analysis of these genes showed that the most mapped functional categories were “general function prediction only”, “posttranslational modification, protein turnover and chaperones”, “transcription” and “signal transduction mechanisms” (**Figure 4A**). Kyoto Encyclopedia of Genes and Genomes (KEGG) enrichment showed that these genes mainly focused on plant hormone signal transduction, plant-pathogen interaction, phenylpropanoid biosynthesis, and MAPK signaling pathway (**Figure 4B**). This indicated that the mechanisms for H_2_S functioning as a gasotransmitter might involve cross-talk with hormone signals and MAPK signals, and regulating the interaction of plant and pathogen as well as the biosynthesis of phenylpropanoid, which has important functions in plant development and plant-environment interaction (Dong and Lin, 2021). Protein-protein interaction (PPI) plays essential roles in many biological processes, and we predicted 24 PPIs in these H_2_S but not Cys regulated DEGs (**Figure 4C**), such as BON1 and BON association protein BAP1, CAF1-9 and CAF1-11, which may provide important insights for revealing H_2_S signaling pathway.

**Figure 5:**
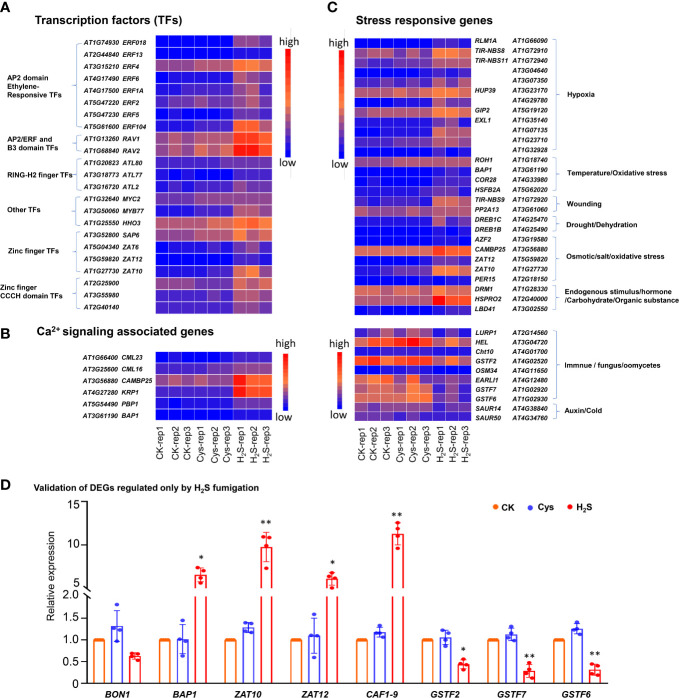
Heatmap representation and qRT-PCR analysis of some DEGs regulated by H_2_S but not by Cys treatment. **(A)** Heatmap representation of the up-regulated DEGs encoding transcription factors. **(B)** Heatmap representation of the DEGs encoding stress responsive genes. **(C)** Heatmap representation of the up-regulated DEGs encoding Ca^2+^signaling associated proteins. **(D)** qRT-PCR analysis of the transcript abundance of *BON1*, *BAP1*, *ZAT10*, *ZAT12*, *CAF1-9*, *GSTF2*, *GSTF7*, and *GSTF6* in H_2_S and Cys treated seedlings. The symbol * indicates significant difference at the 0.05 level (*p* < 0.05) and ** indicates significant difference at the 0.01 level (*p* < 0.01).

In addition, GO enrichment analysis showed that more than half of these H_2_S but not Cys regulated genes encode DNA binding proteins (**Figure 4D**), including AP2 domain ethylene-responsive transcription factors (TFs), AP2/ERF and B3 domain-containing TFs, RING-H2 finger proteins, Zinc finger proteins, Zinc finger CCCH domain-containing proteins, MYB TFs, etc. (**Figure 5A**). Some Ca^2+^ and CaM binding proteins could also be regulated by exogenous H_2_S fumigation, including Ca^2+^-binding proteins, KRP1 (AT4G27280) and PBP1 (AT5G54490), Calmodulin like proteins, CML13 (AT1G66400) and CML16 (AT3G25600), and CaM-binding protein CAMBP25 (AT3G56880) (**Figure 5B**). Furthermore, some stress-responsive genes were also included in these H_2_S but not Cys regulated DEGs, and were represented by the Heatmap (**Figure 5C**). It was indicated that these genes involved in a variety of stress-responsive pathways, including hypoxia, temperature stress, wounding, drought, dehydration, salt and osmotic stress, oxidative stress, etc., also involved in plants defense reactions to pathogen attack as well as response to endogenous signals, such as hormone, carbohydrate, and organic substance (**Figure 5C**).

Furthermore, the expression levels of some DEGs were analyzed by qRT-PCR, and the expression patterns for all chosen mRNAs were consistent with the results based on RNA sequencing (**Figure 5D**). The *BON1* (*AT5G61900*) could be slightly down-regulated by H_2_S fumigation with no statistical difference, but a *BON1*-associated gene *BAP1* (*AT3G61190*) could be significantly up-regulated by H_2_S fumigation (**Figure 5D**). As chloroplast ROS marker genes, both *BAP1* and *ZAT10* (*AT1G27730*) were up-regulated by H_2_S fumigation (**Figure 5D**), indicating that the chloroplasts ROS signaling might be activated by H_2_S signaling. The expression of *CAF1-9* (*AT3G44260*), probable *CCR4-associated factor 1* (*CAF1*) *homolog 9*, was enhanced nearly by 12 times in H_2_S fumigated seedlings (**Figure 5D**). It has been reported that CCR4-CAF1 complex is the major enzyme complex that catalyzes mRNA degradation through initiating mRNA deadenylation (Liang et al., 2009), so the regulation of H_2_S on CAF1-9 might enrich the signal transduction pathway of H_2_S from the perspective of deadenylation induced mRNA degradation. Additionally, three glutathione S-transferase coding genes (*GSTF2*, *GSTF6*, *GSTF7*) were obviously down-regulated by H_2_S fumigation, but not affected by Cys treatment (**Figures 5C, D**).

### 3.6 Analysis of H_2_S and Cys co-regulated DEGs

**Figure 6:**
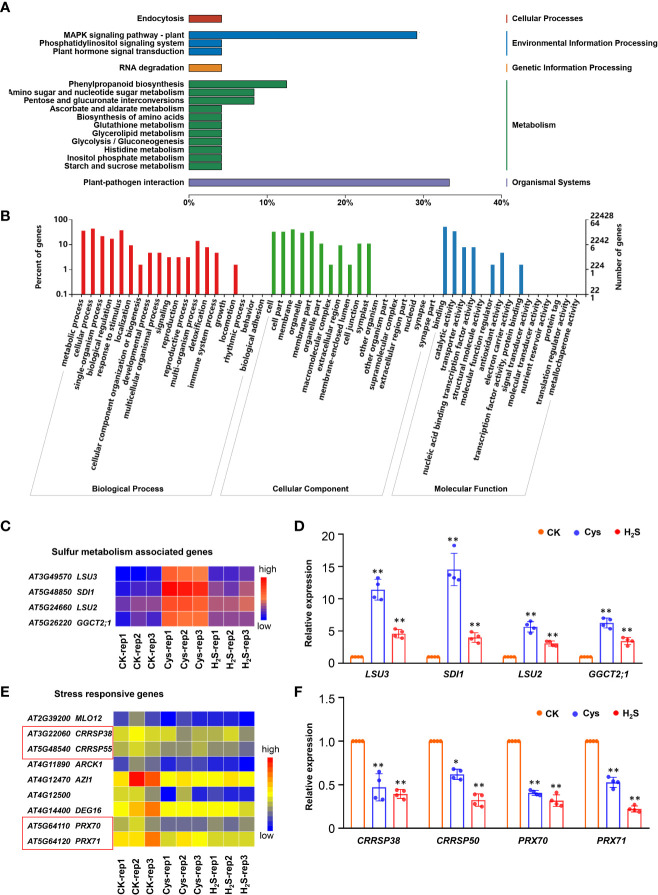
Overall analysis of the DEGs co-regulated by both H_2_S and Cys treatment. **(A, B)** KEGG analysis **(A)** and GO function classification **(B)** of the 72 DEGs that could be co-regulated by both H_2_S and Cys treatment. **(C, D)** Heatmap representation **(C)** and qRT-PCR analysis **(D)** of the sulfur metabolism associated genes (*LSU3*, *SDI1*, *LSU2*, *GGCT2;1*). **(E, F)** Heatmap representation **(E)** and qRT-PCR analysis **(F)** of the stress responsive genes (*CRRSP38*, *CRRSP50*, *PRX70*, *PRX71*). The symbol * indicates significant difference at the 0.05 level (*p* < 0.05) and ** indicates significant difference at the 0.01 level (*p* < 0.01).

Involvement of H_2_S as a substrate in the synthesis of Cys is an important way for H_2_S participating in S metabolism. The H_2_S and Cys co-regulated DEGs might be genes regulated by both H_2_S and Cys signals, or H_2_S-induced Cys signals, or Cys-regulated H_2_S signals, so we analyzed these DEGs to provide evidences for crosstalk between H_2_S and Cys signals pathway. KEGG analysis indicated that the biological functions of these genes mainly involved in environmental information processing (MAPK signaling pathway, Phosphatidylinositol signaling system, Plant hormone signal transduction), organismal system (plant-pathogen interaction), and metabolism pathway (phenylpropanoid biosynthesis, amino sugar and nucleotide sugar metabolism, pentose and glucuronate interconversions, etc.) (**Figure 6A**). GO function classification indicated that these genes involved in 34 subcategories of biological processes, cellular components, and molecular functions. For the biological process, “cellular process”, “metabolic process”, and “response to stimulus” were the most representative groups. In the subcategory of cellular components, “membrane”, “cell part”, “membrane part” were the predominant groups. For the molecular function, “binding” and “catalytic activity” were the most common (**Figure 6B**).

Sulfur is essential for all living organisms on earth as a key component of amino acids (i.e., Cys and methionine), polypeptide glutathione, several group transfer coenzymes and vitamins (Romero et al., 2014). An in-depth study of these genes showed that the transcription of some S metabolism associated genes (*LSU2*, *LSU3*, *SDI1*, and *GGCT2;1*), which involve in response to low sulfur and sulfur deficiency, were significantly increased in both H_2_S and Cys treated seedlings (**Figures 6C, D**), suggesting that the treatment of exogenous S compounds (H_2_S and Cys) might regulate S absorption and metabolism (Sirko et al., 2014; Garcia-Molina et al., 2017), which might involve the feedback regulation of S absorption and metabolism. H_2_S-Cys cycle may participate in the feedback regulation of S-containing compound on S response. Some stress responsive genes could be down-regulated by both H_2_S and Cys treatments, including *MILDEW RESISTANCE LOCUS O* (*MLO12*), cysteine-rich receptor-like secreted protein encoding genes (*CRRSP38* and *CRRSP55*), and the peroxidase superfamily genes (*PRX70* and *PRX71*), this might be attribute to the enhancement of endogenous Cys and Cys-related signaling pathways (**Figures 6E, F**). The expression changes of some DEGs in H_2_S and Cys treated seedlings detected by qRT-PCR were consistent with the results from RNA sequencing data (**Figures 6D, F**), which further validated the reliability of the high-throughput sequencing results in this study.

### 3.7 Activation of anthocyanin synthesis induced by Cys but not H_2_S treatment

In plants, anthocyanins play a role not only in reproduction, by attracting pollinators and seed disseminators, but also in protecting plants from various abiotic and biotic stresses. More and more evidences show that anthocyanins have health-promoting properties, which makes anthocyanin metabolism an interesting target for breeders and researchers (Pojer et al., 2013; Liu et al., 2018). Anthocyanins are synthesized from 4-coumaroyl-CoA, which is generated from phenylalanine *via* the general phenylpropanoid pathway, involving the phenylalanine ammonia lyase (PAL), cinnamate 4-hydroxylase (C4H) and 4-coumaryol CoA ligase (4CL). One molecule of 4-coumaroyl-CoA and three molecules of malonyl-CoA are condensed by chalcone synthase (CHS), which is the starting reaction of flavonoid biosynthesis. A coloured anthocyanidin (pelargonidin) are synthesized through sequential enzyme reactions involving the chalcone isomerase (CHI), flavanone 3-hydroxylase (F3H), dihydroflavonol 4-reductase (DFR) and anthocyanidin synthase (ANS, also known as LDOX), and UDP-glucose: flavonoid glucosyltransferase (UFGT) (**Figure 2A**).

Our data indicated that the anthocyanin level of the leaves was increased by Cys treatment but not affected by H_2_S fumigation (**Figure 2B**), which consistent with the phenotype that darker anthocyanin color in leaves of Cys-treated seedlings, especially in the petioles and center veins of leaves (**Figure 1B**). Both the RNA sequencing and the qRT-PCR data indicated that these anthocyanin-synthesis-associated genes (*CHS*, *CHI*, *F3H*, *DFR*, *LDOX*, *UF3GT*) were significantly up-regulated by Cys treatment, but had no obvious changes in H_2_S fumigated seedlings (**Figures 2C, D**), indicating that Cys enhanced anthocyanin level through up-regulating the expression of genes responsible for anthocyanin synthesis. The expression of *DFR*, encoding the key enzyme in the anthocyanin biosynthetic pathway, was even increased nearly sevenfold in Cys treated seedlings (**Figure 2D**). H_2_S fumigation enhanced endogenous Cys content but didn’t promote anthocyanin accumulation, which suggested the distinction of H_2_S and Cys signals, indicating that H_2_S could regulates biological processes as a gasotransmitter in a Cys-independent manner.

## 4 Discussion

H_2_S is well-known because of its unpleasant odor and high toxicity, and the toxicological properties of H_2_S were focused in most of the early researches (Wang, 2002; Fu et al., 2018). Until recent decades, increasing evidence revealed that H_2_S functions as a signaling molecule in plant growth and development as well as in biotic and abiotic stress responses (Wang, 2014; Paul and Snyder, 2015; Aroca et al., 2018; Liu et al., 2021; Zhang et al., 2021; Li et al., 2022; Yang et al., 2022). However, some scientists remain skeptical about its direct role as gasotransmitter and maintain that the positive function of H_2_S may largely depend on S-containing organic molecules, especially Cys, which has been demonstrated to play roles in various cellular processes (Paulsen and Carroll, 2013; Romero et al., 2014). During S metabolism in plants, H_2_S, uptaken directly from the atmosphere or generated form sulfate, could act as the substrate to generate Cys, while Cys can be degraded to produce H_2_S, so the changes of H_2_S level will undoubtedly influence Cys content and metabolism. Therefore, revealing the difference and relationship between H_2_S signaling and Cys-related signaling will be of great significance for clarifying the gasotransmitter functions of H_2_S.

Based on our study, H_2_S fumigation obviously increased both the endogenous H_2_S and Cys content, meanwhile Cys treatment enhanced endogenous Cys and H_2_S content to various degrees (**Figures 1C, E**), indicating that the endogenous H_2_S and Cys signals could be activated by exogenous H_2_S and Cys treatments. In addition, based on our transcriptome data, we analyzed and compared the FPKM value of genes associated Cys synthesis and degradation, including *AtOAS-A1*, *AtOAS-B*, *AtOAS-C*, *AtCYS-C1*, *AtCYS-D1*, *AtCYS-D2*, *AtCS26*, *AtDES1*, *AtLCD*, *AtDCD1*, *AtDCD2*, *AtNFS1*, and *AtNFS2*, in Control, Cys-treated, and H_2_S-fumigated seedlings, and data indicated that both Cys and H_2_S treatment can not affect the expression of these genes obviously (**Table S2**). Therefore, it might be speculated that Cys or H_2_S might regulate Cys and H_2_S metabolism by regulating the activities of corresponding enzymes rather than the expressions of corresponding genes.

Comprehensive transcriptome analysis indicated that H_2_S and Cys treatment can induced different DEGs in seedlings, and there were 189 genes could be regulated only by H_2_S fumigation but not response to Cys treatment (**Figure 3H**), hinting that H_2_S involves in regulating gene transcription through its gasotransmitter function, rather than only as a substrate of Cys, and there is a high probability that these 189 genes function importantly in H_2_S signal transduction pathways.

It has been reported that H_2_S interacts widely with phytohormones in diverse processes during the developmental and environmental responses of plants. H_2_S treatment down-regulated the ethylene biosynthesis genes, while up-regulated the ethylene receptor genes in banana fruit (Ge et al., 2017). H_2_S induced persulfidation on ACOs to inhibit ethylene synthesis involving osmotic stress response (Jia et al., 2018). The endogenous biosynthesis and/or signaling of ethylene and auxin could be modulated by H_2_S to inhibit the process of petiole abscission in tomato (Liu et al., 2020). Moreover, H_2_S might act as a downstream component of auxin signaling to trigger lateral root formation in tomato (Fang T, et al., 2014). The interaction between H_2_S and ABA signaling has been well studied in regulating stomatal movement. As early as 2012, Jin et al. reported that H_2_S interacting with ABA in the stomatal regulation responds to drought stress in Arabidopsis (Jin et al., 2013). Recently, it was found that during stomatal response to ABA induction, H_2_S mediated DES1 persulfidation to amplify H_2_S signal, which then drive the persulfidation of the NADPH oxidase RBOHD to produce ROS and induce stomatal closure (Shen et al., 2020). H_2_S induces the persulfidation of SnRK2.6/OST1 to positively regulate ABA signaling mediated stomatal movement (Chen et al., 2020). H_2_S mediated the persulfidation of ABI4, a key positive regulator of ABA signaling, to improve its ability for activating *MAPKKK18* transcription, which is important for ABA response in Arabidopsis (Zhou et al., 2021). Above all, it seems that H_2_S functions not only as an integral molecule in the hormone signaling network of plants but also as a “referee” to harmonize the interaction between hormones. Therefore, revealing the detailed mechanism of the interactions between H_2_S and these phytohormones during the respective physiological processes will be valuable. Focusing on these 189 genes, regulated only by H_2_S fumigation but not Cys treatment, we found that many of these genes involve in plant hormone signal transduction (**Figure 4B**), including ethylene responsive TFs, auxin-responsive genes, salicylic acid responsive genes, ABA signaling associated genes, etc. (**Figure 5A**). Moreover, more than half of these genes encoded protein providing DNA binding and transcription factor activities (**Figures 4D**, **5A**), participating or having crosstalk with many phytohormones signals. Our transcriptome data indicated that these 189 genes might function as the alternative downstream components in H_2_S signal transduction and crosstalk with hormone signal pathways, providing some insights for determining the mechanism of H_2_S interaction with phytohormones.

H_2_S also interacts with Ca^2+^ and calcium signaling, especially in the process of plant response to metal stress. Our previous study indicated that Ca^2+^/CaM2 physically interacts with bZIP transcription factor TGA3 to enhance its ability for activating *LCD* transcription, then improve the production efficiency of endogenous H_2_S in *Arabidopsis* response to Cr^6+^ stress (Fang et al., 2017). Similarly, H_2_S dependent pathway is also a component of Ca^2+^ mediated activation of the antioxidant system and upregulation of the expression of genes associating with heavy metal chelation in *Setaria italica* coping with Cr^6+^ stress (Fang H, et al., 2014). Additionally, H_2_S can strongly enhanced Ca^2+^ induced upregulation of *CaM* and *CBL* expressions in *Setaria italica* under Cr^6+^ stress (Fang H, et al., 2014). NaHS treatment increased the *CDPK* transcripts in seedling leaves of zucchini under Ni stress (Valivand et al., 2019), while CDPK3 enhanced LCD activity and intensified H_2_S signal to enhance the tolerance of Arabidopsis to Cd stress (Qiao et al., 2016). These researches suggest that H_2_S and Ca^2+^ signal do not have a simple upstream and downstream relationship, but a complex cross interaction in plants response to metal stress. In this study, some Ca^2+^ signal transduction associated genes (*CML23*, *CML16*, *CAMBP25*, *KRP1*, *PBP1* and *BAP1*) could be upregulated by H_2_S fumigation (**Figure 5B**). *BAP1*, encoding a membrane-associated protein containing a Ca^2+^-dependent phospholipid-binding C2 domain, involves in programmed cell death (PCD) and defense reaction across the kingdoms (Yang et al., 2007), so the upregulation of H_2_S fumigation on this gene strongly hinted the function of H_2_S in regulating PCD and defense, and the underlying mechanism is probably related to the interaction with Ca^2+^ signaling.

H_2_S has been widely reported to participate and/or regulate the ROS signal transduction, such as activating some antioxidant enzymes through inducing persulfidation, increasing GSH content, interacting with H_2_O_2_ signal, etc. (Choudhary and Chaudhary, 2021; Liu et al., 2021; Zhang et al., 2021). Chloroplast ROS signaling is a major driving force in chloroplast to nucleus retrograde signaling and plays important roles in plant stress responses (Foyer and Hanke, 2022; Li and Kim, 2022). Based on our data in this study, the chloroplast ROS marker genes *ZAT10* and *BAP1* were up-regulated by H_2_S fumigation, but not affected by Cys treatment, indicating that H_2_S might induce the chloroplast ROS signaling independently of Cys.

Comprehensively, our results provide evidence for H_2_S as a gasotransmitter besides as the substrate for Cys synthesis in regulating plant response to both developmental and environmental cues, and further enrich and deepen the H_2_S signal transduction networks. However, the underlying mechanism of H_2_S mediating the transcriptional regulation of these DEGs is still worth of further exploration. H_2_S-induced persulfidation has been proved to be an important post-translational modification (PTM) in plants (Aroca et al., 2018; Gotor et al., 2019; Chen et al., 2020; Ma et al., 2021; Zhou et al., 2021), so H_2_S might regulating genes expression through inducing persulfidation on proteins that function as transcription factors or involve in transcriptional regulatory complexes of target genes. Determining the connection between H_2_S mediated regulation of protein persulfidation and gene transcription is very significant for revealing the detailed mechanism of H_2_S signal transduction.

## Data availability statement

The transcriptomic data presented in the study are deposited in the National Center for Biotechnology Information (NCBI) repository, and the BioProject accession number PRJNA971675.

## Author contributions

HF conceived the present idea and wrote the manuscript. ZY, KX, and LZhou performed the experiment most. HF, ZY, and KX performed the data analysis, HF, YP and LZhang supervised the project, provided critical feedback and helped shape the final manuscript. All authors helped perform the analysis with constructive discussions, and proofread the manuscript. All authors contributed to the article and approved the submitted version.

## Supplementary material

Supplementary Figure 1 Analysis of transcriptome profiles in seedlings responding to H_2_S and Cys treatment. **(A, B)** Boxplot and density distribution diagram showing the gene expression features in 9 transcriptome samples of CK, H_2_S fumigated and Cys treated seedlings. FPKM, fragments per kilobase million. **(C)** Principal component analysis (PCA) of gene expression in 9 transcriptome samples of CK, H_2_S fumigated and Cys treated seedlings.
