# Supplementary figures and images for: Transcriptomic analysis reveals the functions of H2S as a gasotransmitter independently of Cys in Arabidopsis
Source: Front Plant Sci. 2023 Jun 2;14:1184991. doi: 10.3389/fpls.2023.1184991 (PMC10272727; doi:10.3389/fpls.2023.1184991)

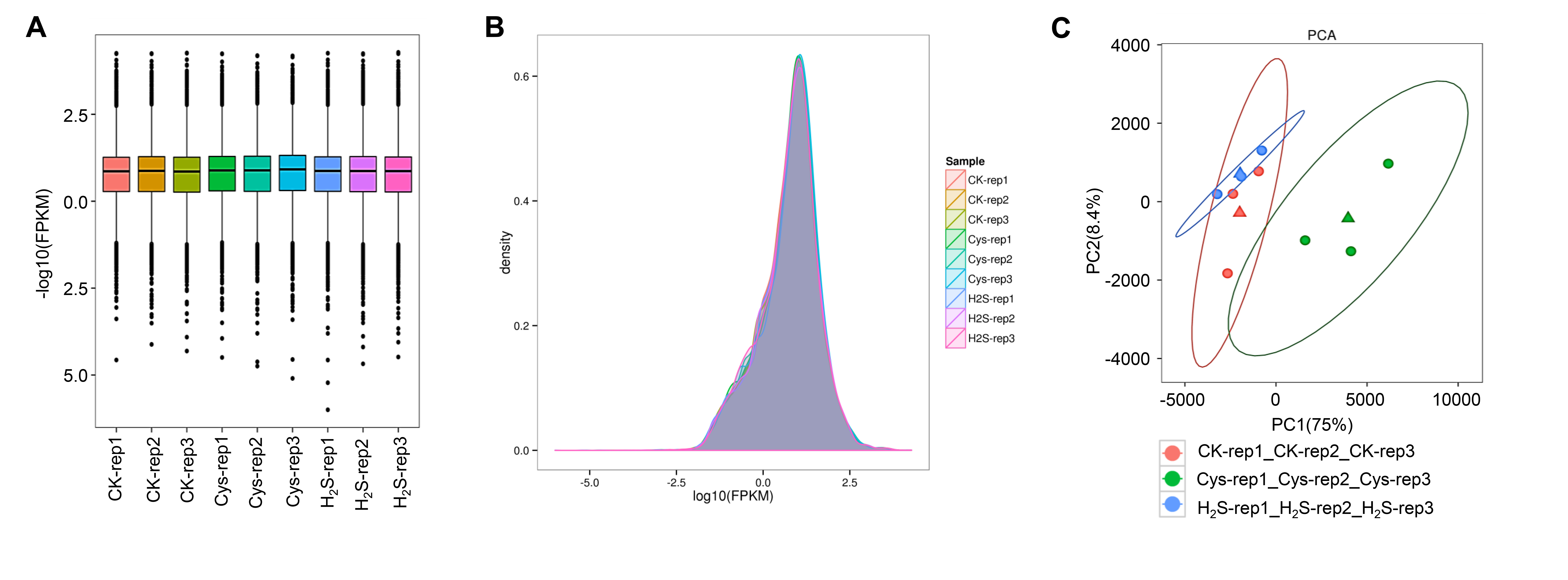

Supplement: Supplementary Figure 1 — Analysis of transcriptome profiles in seedlings responding to H2S and Cys treatment. (A, B) Boxplot and density distribution diagram showing the gene expression features in 9 transcriptome samples of CK, H2S fumigated and Cys treated seedlings. FPKM, fragments per kilobase million. (C) Principal component analysis (PCA) of gene expression in 9 transcriptome samples of CK, H2S fumigated and Cys treated seedlings. [file DataSheet_1.zip › Figure S1.JPEG]
